# Functional near-infrared spectroscopy (fNIRS) in medical research: applications, opportunities, and challenges

**DOI:** 10.1186/s13020-026-01409-w

**Published:** 2026-06-18

**Authors:** Qingya Yu, Qiuxu Yuan, Yuying Song, Nana Feng, Yuanyuan Li, Wenbin Liu, Huanang Chen, Zhimin Zhou, Shaofeng Shuai, Ce Tang, Dingkun Zhang, Yi Zhang, Caiyun Zhao

**Affiliations:** 1 Chinese Medicine Germplasm Resources Innovation and Effective Uses Key Laboratory of Sichuan Province, School of Pharmacy, Chengdu University of Traditional Chinese Medicine, Chengdu, 611137 China; 2 School of Ethnic Medicine, Chengdu University of Traditional Chinese Medicine, Chengdu, 611137 Sichuan China; 3 School of Modern Chinese Medicine Industry, Chengdu University of Traditional Chinese Medicine, Chengdu, 611137 Sichuan China; 4 School of Traditional Dai Medicine, West Yunnan University of Applied Sciences, Jinghong, 666100 Yunnan China

**Keywords:** Functional near-Infrared Spectroscopy (fNIRS), Pharmaceuticals, Traditional Chinese medicine, Brain functional imaging, Clinical application

## Abstract

fNIRS is an innovative and non-invasive tool for monitoring hemodynamic changes in the cerebral cortex. In recent years, it has become a widely used intervention method in the medical field for treating various diseases due to its portability and potential for long-term monitoring. This article systematically reviews the development history and technical principles of fNIRS, compares fNIRS with other imaging techniques, summarizes the application and research progress of fNIRS in the fields of neurofunction and brain function, clinical medicine, traditional Chinese medicine (TCM), and rehabilitation medicine, especially in exploring the neurobiological mechanisms of TCM syndromes, monitoring the central effects of acupuncture therapy, and evaluating the brain's response to TCM components. At the same time, it clarifies the shortcomings and deficiencies of fNIRS and discusses its future development, aiming to provide valuable insights and reference points for the future development of fNIRS.

**Graphical Abstract:**

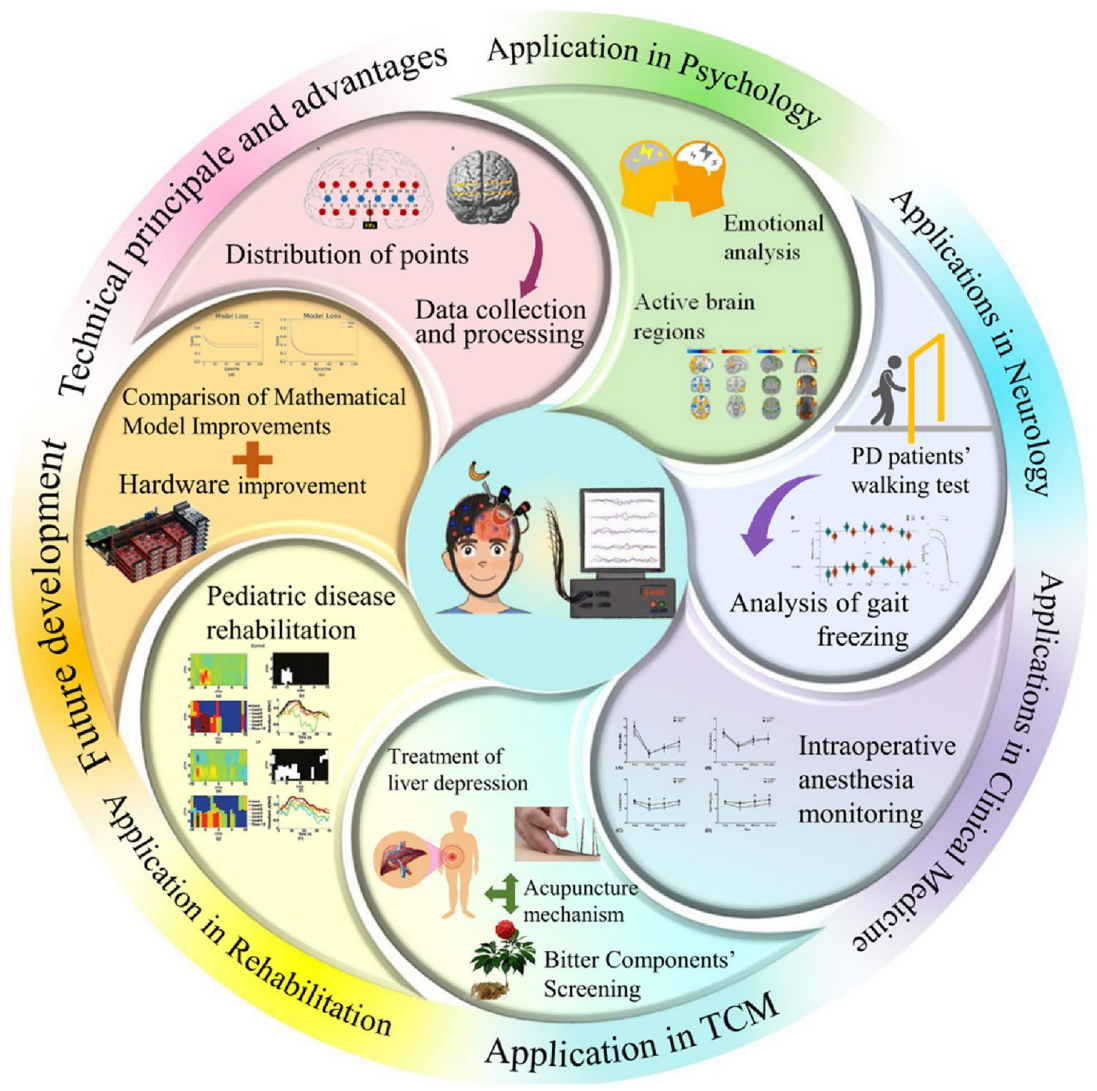

## Introduction

fNIRS is a non-invasive brain functional imaging method based on near-infrared optics [1]. This technique uses near-infrared light with wavelengths of 650–950 nm to illuminate the scalp. This wavelength range is known as the "optical window", and its physical basis lies in the fact that within this wavelength range, oxygenated hemoglobin (HbO_2_) and deoxygenated hemoglobin (HbR) exhibit distinct and distinguishable absorption characteristics. Meanwhile, water, which is highly abundant in human tissues, shows significantly enhanced absorption at wavelengths above 950 nm, and lipids, which absorb more at shorter wavelengths, have relatively low absorption in this near-infrared band. This allows the incident light to effectively penetrate the scalp, skull, and superficial brain tissue, reaching the cerebral cortex. By measuring the attenuation changes of light in the tissue, fNIRS can monitor the real-time changes in the concentrations of HbO_2_ and HbR, thereby indirectly reflecting local neural activity based on the neurovascular coupling mechanism [1, 2]. Compared with traditional brain imaging techniques such as functional magnetic resonance imaging (fMRI) and positron emission tomography (PET), fNIRS overcomes their strict limitations on environment, body position and the cooperation of subjects. It has the advantages of high portability, strong resistance to motion interference, good ecological validity and low cost. It is especially suitable for brain function research on infants, the elderly, clinical patients and in natural settings [3, 4].

In recent years, with the continuous development of hardware equipment and data analysis methods, fNIRS has been increasingly widely applied in various fields such as neuroscience, psychology, clinical medicine, and TCM in research [5, 6]. Although the research results of fNIRS technology in the medical field have been increasing year by year, the existing reviews mostly focus on a single application direction (such as neurodegenerative disease assessment or cognitive function research), and lack a systematic integration and comprehensive review of the application in neuroscience, clinical medicine and TCM. Especially in the field of TCM, although the research using fNIRS to explore the neurobiological mechanism of TCM syndromes, monitor the central effect of acupuncture therapy and evaluate the brain response to Chinese medicine components has shown an increasing trend, it is still at the stage of method exploration and lacks systematic summarization of existing results and methodological reflection. In addition, fNIRS technology still faces technical bottlenecks such as signal processing, data standardization and individual differences in practical applications, which restricts its clinical transformation process. Therefore, systematically integrating the research progress of fNIRS in multiple medical fields and clarifying the current technical limitations and development directions have important academic value and practical significance.

Based on the above background, this paper systematically retrieved relevant literature from electronic databases such as SCI Finder, PubMed, Web of Science, Science Direct, Springer, and Google Scholar, covering the period from 1980 to 2025. Using keywords like "fNIRS", "functional near-infrared spectroscopy", "brain function", "clinical application", and "TCM", the paper screened out representative research results highly relevant to the topic by reading the titles and abstracts. Based on the literature review, this paper first reviewed the development history and technical principles of fNIRS and compared it with other imaging technologies; subsequently, it focused on summarizing the research progress of fNIRS in neuroscience, clinical medicine, TCM, and rehabilitation medicine, particularly emphasizing its applications in cognitive function assessment, diagnosis of neurological diseases, research on mental disorders, exploration of the mechanism of TCM syndromes, monitoring of acupuncture effects, and screening of bitter components of TCM; finally, taking into account the current technical limitations, the development prospects of fNIRS in the fields of individualized medicine, drug research and development, and the integration with artificial intelligence were discussed. Among them, the potential of fNIRS in the screening of neurobiological markers, drug screening, and rehabilitation assessment mainly stems from its portability, real-time monitoring capability, and the quantifiable characteristics of brain functional activities in natural contexts. Existing studies have provided preliminary evidence in areas such as optimizing neurorehabilitation training programs, monitoring changes in cerebral hemodynamic under drug intervention, and evaluating individual differences in patients' treatment responses [7–9]. Through the above systematic review, this article aims to fill the gap in cross-domain integration of existing reviews, provide a systematic reference for in-depth research and application transformation of fNIRS technology in the medical field, and promote its further development in precision medicine and modernization research of TCM.

### Development and principles of fNIRS technology

**Fig. 1:**
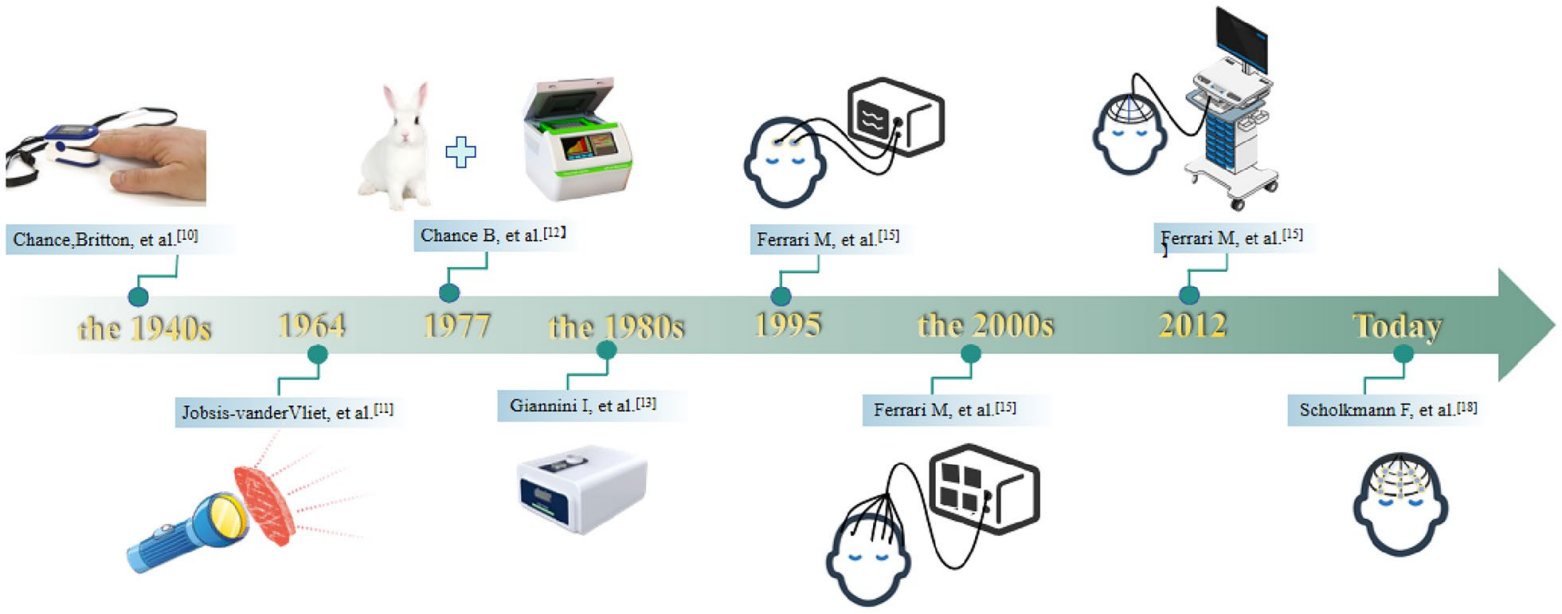
Timeline of the development of fNIRS

The development of optical methods can be traced back to the muscle oximeter invented by Glenn Millikan in the 1940s [10]. In 1964, Frans Jöbsis discovered that when visible light was blocked by tissue samples, red light could penetrate the samples, indicating that red light and even longer near-infrared light could better pass through animal tissues and bones to reach the underlying tissues. This laid the foundation for the development of near-infrared light in the medical and biological fields [11]. In 1977, he reported that the relatively high transparency of brain tissue in the near-infrared range enabled realtime noninvasive detection of hemoglobin oxygenation using trans-illumination spectroscopy. As a result, Frans Jöbsis is regarded as the founder of in vivo near-infrared spectroscopy [12]. In 1980, Marco Ferrari utilized prototype near-infrared spectroscopy to measure changes in cerebral oxygenation in experimental animals. In 1985, data on the effects of carotid compression on regional cerebral oxygenation and volume in cerebrovascular patients, as well as cerebral measurements in newborns, were presented at an academic conference [13]. In 1984, David Delpy began to develope several NIRS instruments, and 3 years later, reported the first quantitative measurements of various oxygenation and hemodynamic parameters in critically ill newborns, including concentrations of HbO₂, HbR, total hemoglobin (tHb), cerebral blood volume, and cerebral blood flow. This work significantly advanced the development of fNIRS [14]. From 1980 to 1995, institutions such as the University of Zurich and the University of Pennsylvania collaborated with nine companies to participate in the development of near-infrared spectroscopy prototypes, accelerating the dissemination and application of this technology [15]. Figure 1 presents the development process of functional near-infrared technology and some key milestones.

fNIRS is a noninvasive imaging technique based on spectroscopic principles, primarily used to monitor cerebral Blood oxygen level changes. Metabolic activities involving blood supply provide oxygen necessary for neurons in the brain, with oxygen transport primarily relying on hemoglobin in the blood. When oxygen in the blood is consumed, it stimulates local vasodilation in the brain, leading to increased capillary blood flow. This results in elevated regional cerebral blood flow (rCBF) and cerebral blood volume (CBV), rapidly enhancing cerebral oxygen levels—a process known as neurovascular coupling [16]. The functionality of fNIRS is fundamentally based on this neurovascular coupling mechanism [17]. Under this mechanism, neural activity increases oxygen consumption [18], and the oxygen delivered by the increased cerebral blood flow in active brain regions far exceeds the oxygen demand required for neural activity. Consequently, during cognitive processes, neural activation leads to a rise in HbO₂ concentration and a decrease in HbR concentration [19]. These processes induce changes in optical attenuation within regional tissues, which can be detected by fNIRS and serve as indirect indicators of brain region activation [1].

The fundamental physical principle of fNIRS is to utilize the penetration capability of near-infrared light with wavelengths of 650–950 nm in biological tissues to monitor concentration changes of HbO₂ and HbR hemoglobin in the blood [20, 21]. The essence of light absorption is the conversion of photon energy into internal energy within the propagating medium. Human biological tissues contain multiple endogenous optical chromophores, primarily including water, lipids, HbO_2_, and HbR, each exhibiting characteristic absorption spectra across different wavelengths [18]. In the visible light range (400–600 nm), tissue absorption is relatively high, resulting in shallow light penetration depth. In contrast, within the 600–950 nm wavelength band, the absorption coefficients of water and lipids remain low, while hemoglobin still exhibits detectable characteristic absorption, with light scattering dominating. This allows near-infrared light to effectively penetrate the scalp, skull, and cortical tissue, defining this range as the near-infrared optical window [18, 21]. Within this optical window, water absorption increases gradually with rising wavelength, and lipid absorption is relatively prominent in the shorter near-infrared region; together, these constitute the background tissue absorption. As the core chromophores underlying neural activity signals, HbO_2_ and HbR show distinct absorption profiles: HbO_2_ exhibits stronger absorption at wavelengths above 850 nm, whereas HbR shows stronger absorption below 850 nm. The 850 nm wavelength represents the isosbestic point, where the molar absorption coefficients of the two chromophores are approximately equal [18, 20]. This differential absorption characteristic forms the physical basis for fNIRS to distinguish and quantitatively detect changes in HbO_2_ and HbR concentrations [18, 19].

### Advantages and characteristics of fNIRS technology

**Fig. 2:**
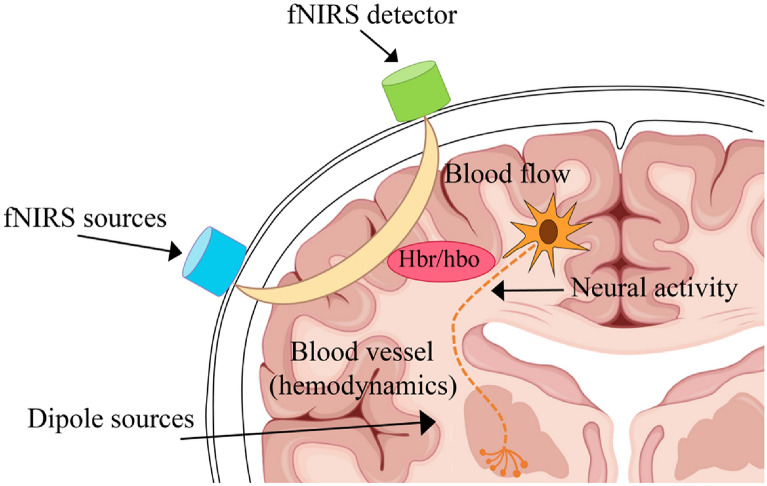
Propagation path of near-infrared light in a near-infrared detection system

A typical fNIRS system consists of light sources and photodetectors. Light sources generally employ laser diodes or light-emitting diodes to deliver near-infrared light of specific wavelengths into the scalp; photodetectors typically use photodiodes to capture light signals exiting the tissue after attenuation. Each pair of source and detector forms a measurement “channel”. The spatial position and length of the channel determine the depth and coverage of the probed brain region. Longer channels allow light to penetrate deeper; however, increasing the distance between the source and detector elevates the signal-to-noise ratio and may result in signal loss [22, 23]. The probabilistic path of NIR light propagation (i.e., the path length of light) is longer than the actual channel distance, as some light can penetrate the tissue, forming a so-called "banana-shaped" optical path [15]. Figure 2 illustrates the propagation path of functional near-infrared during the detection process. Compared with other imaging modalities, fNIRS offers unique advantages. First, fNIRS devices are relatively portable and easy to operate, making them suitable for both clinical and non-clinical environments [24]. Second, fNIRS exhibits greater tolerance to motion artifacts, making it suitable for populations who may have difficulty remaining still, such as children and elderly patients [25]. Additionally, fNIRS is relatively low-cost and enables real-time monitoring, which is often challenging to achieve with fMRI and EEG [2]. However, fNIRS has moderate spatial resolution and limited penetration depth, which restricts its application in studying certain complex brain activities [26]. Nevertheless, its high temporal resolution makes it widely useful in various clinical studies, such as research on depression and bipolar disorder [27]. Furthermore, fNIRS can measure a wide range of data, including functional comparisons of HbO₂, HbR, and tHb. Compared with fMRI, fMRI measures the blood oxygen level dependence, while fNIRS measures the nonlinear function of oxygen levels and cerebral blood flow [28]. Additionally, the relatively high spatial resolution of fNIRS enhances its utility in functional connectivity analysis, making it a new and increasingly adopted neuroimaging paradigm (Table 1) [29–32].

**Table 1:** Comparison of parameters of commonly used brain imaging techniques

Comparison dimension	fNIRS	fMRI	EEG	MEG	PET	References
Spatial resolution	Centimeter-level (1–3 cm), the equivalent diameter of the measurement area determined by the scattering path of photons within the tissue	At the millimeter level (1–3 mm), it reflects the spatial positioning accuracy of the blood oxygen level-dependent (BOLD) signal at the three-dimensional voxel level. It has a different physiological basis and spatial scale from the signals measured by fNIRS and should not be directly compared in terms of spatial resolution	Centimeter-level (1–2 cm), with significant volume conduction effect, but poor spatial source localization accuracy	Millimeter-scale (3–5 mm), with minimal interference to the magnetic field signal and superior spatial positioning compared to EEG	Millimeter-level (2–4 mm), capable of achieving precise imaging of brain metabolism and blood flow throughout the entire brain	[18, 33, 34]
Temporal resolution	10–100 ms, capable of capturing rapid hemodynamic responses, lagging behind neural electrical activity by approximately 1–2 s	1–2 s, due to the limitation of neural-vascular coupling delay, it is impossible to track neural electrical activity in real time	1–10 ms, real-time recording of neuronal electrical activity at the millisecond level, with the highest temporal resolution	1–10 ms, consistent with EEG, can precisely capture the temporal characteristics of neural electrical activities	Minute-level (3–5 min), requiring accumulation of tracer metabolism, with extremely low temporal resolution	[2, 15, 18]
Signal foundation	Based on the near-infrared light absorption characteristics, the concentrations of HbO, HbR, and HbT are detected to reflect the cerebral hemodynamic response	Based on BOLD effect, neural activity is reflected through the magnetic susceptibility difference of deoxygenated hemoglobin	Record the electrical signals generated by the postsynaptic potentials of neurons in the cerebral cortex, which directly reflect the neural electrical activity	Detect the weak magnetic field signals generated by neuronal electrical activities, which directly reflect the spatiotemporal characteristics of neural electrical activities	By using radioactive nuclide-labeled tracers, the glucose metabolism of brain tissue, local cerebral blood flow and receptor distribution can be reflected	[15, 33, 35, 36]
Investigation depth	1–2 cm, capable of detecting only the superficial areas of the cerebral cortex, unable to detect the subcortical nuclei	Fully covers the entire brain, capable of precisely detecting deep brain regions such as the cortex, subcortical nuclei, brainstem, and cerebellum	0.5–1 cm, only recording superficial cortical electrical signals, with severe attenuation of signals in deep brain regions	1–3 cm, capable of detecting the cortical and some mid-layer brain regions, with superior magnetic field signal penetration compared to electrical signals	Full brain coverage, capable of detecting metabolic and blood flow information in all brain regions including the cortex, subcortex, and brainstem	[18, 37, 38]
Invasive/Noninvasive	Completely non-invasive. The optical probe is in contact with the scalp. No radiation, no trauma, no adverse reactions	Completely non-invasive, magnetic field imaging, no radiation, no invasive procedures	Completely non-invasive. The electrodes are attached to the scalp. No radiation, no trauma	Completely non-invasive, magnetic field detection, no radiation, no invasive operation	Invasive procedure, requiring intravenous injection of radioactive tracer, with the risk of ionizing radiation exposure	[34, 35, 39]
Anti-motion interference capability	Strong, the optical signal is less affected by slight head movements, and can be used for dynamic tasks such as active movement and walking	The range of variation is so small that even a slight movement of the head can cause severe artifacts. It is only applicable to tasks performed in a resting or fixed position	Weaker, head movement and electromyographic interference are prone to cause artifacts, so the head needs to be firmly fixed	The range of variation is extremely sensitive to head movement. The position of the head must be strictly fixed	The range of variation indicates that movement can cause uneven distribution of the tracer, and absolute stillness must be maintained during imaging	[8, 37, 40]
Portability	High, the equipment is lightweight and wearable, and it supports detection in various natural scenarios such as bedside, outdoor, and clinical rehabilitation	Extremely low, large-scale fixed equipment, requires a dedicated shielding room, and is only applicable in laboratory environments	Medium level. The equipment is portable but the electrode wiring is complex. It can be used in scenarios such as bedside and sleep monitoring	Extremely low, requires a superconducting magnetic shielding chamber. The equipment is bulky and is only suitable for professional laboratories	Extremely low, large-scale nuclear medical equipment is permanently installed in hospitals and cannot be moved	[38, 40, 41]
Metal compatibility	Compatible, applicable for patients with metal implants (such as titanium plates, pacemakers) for detection purposes	Incompatibility. Strong magnetic fields can cause metal implants to shift and heat up, posing safety risks	Compatible, unaffected by metal implants, with wide application range	Incompatible. Magnetic field interferes with metal implants. Prohibited for use in patients with metal implants	Compatible, without magnetic field interference, suitable for patients with metal implants	[37, 39, 40]
Scanning duration	Short, in the resting state, ≥ 4 min is sufficient to obtain stable and reliable data. The task state can be completed within a few minutes	Longer, with a single scan taking 5–10 min. Whole-brain imaging requires a much longer time	Short, the signal collection can be completed within seconds to several minutes, and real-time monitoring is available	Longer, single scan takes 5–10 min, and the head position needs to be stable	Long, the metabolism of the tracer agent takes 10–30 min. The scanning process is time-consuming and inefficient	[15, 33, 42]
Equipment cost	Low–medium, with affordable equipment prices and low maintenance costs, suitable for widespread application	Extremely high. The costs for equipment procurement, maintenance, and site construction are very high	Low, the equipment is inexpensive and easy to operate, making it suitable for both grassroots and large-scale research	Extremely high. The construction costs of superconducting equipment and shielding chambers are extremely high, and only a few institutions have them	Extremely high. The cost of nuclear medical equipment and tracers is high, and strict radiation protection requirements are necessary	[35, 38, 41]
Operational threshold	Low, the probe is easy to wear and does not require professional training. Data collection can be completed quickly	Highly complex, requiring professional technicians to operate. The requirements for the subjects' cooperation are also very high, and the data processing is extremely complicated	Moderate. The electrode positioning needs to be standardized. The removal of artifacts and data analysis are somewhat challenging	Extremely high. The construction costs of superconducting equipment and shielding chambers are extremely high, and only a few institutions have them	Extremely high. It requires operation by nuclear medicine professionals. Radiation protection and trace agent management must be strictly adhered to	[8, 39, 41]
Test–retest reliability	High, the hemodynamic indicators in the resting state and task state show good stability, with ICC ≥ 0.5	High, with excellent stability of the whole brain functional signals and good retest consistency	Medium. The electrical signals are easily affected by the state and interference, and the retest stability is average	High, the magnetic field signal is stable, and the consistency of the re-measurement is better than that of EEG	Moderate. The retest stability is affected by the metabolism of the tracer and individual differences	[33, 42, 43]
Core advantage	High ecological validity, resistance to motion interference, portability and mobility, friendly for children/clinical patients, low cost	High spatial resolution of the entire brain, integrated functional and structural features, and stable and precise signals	High time resolution, non-invasive, low cost, real-time monitoring, wide range of applicable scenarios	The electrical signals have precise traceability, no volumetric conduction, and superior spatial positioning compared to EEG	Quantitative whole-brain metabolism, receptor imaging, with high specificity for disease diagnosis	[15, 18, 36]
Core limitation	Only detects the superficial epidermis layer, has limited spatial resolution, and the signal is easily interfered with by scalp blood flow	Limited movement, claustrophobia, immovable, metal incompatible	Poor spatial positioning, severe volume conduction, and significant electromyography / electrooculography artifacts	Extremely high cost, limited scenarios, sensitive to movement, and low penetration rate	Ionizing radiation, invasiveness, poor durability, scarcity of equipment	[35, 38, 43]

### Unique advantages in the pharmaceutical

fNIRS can stably acquire brain functional signals when the subjects maintain natural behavioral states (such as sitting, standing, and walking) and perform dynamic tasks like active speech and limb movements, thus breaking through the strict limitations of traditional neuroimaging techniques on the subjects' body positions and head stillness. It provides a feasible solution for brain function research with high ecological validity. Compared with techniques like fMRI, fNIRS does not require strict constraints on the subjects' body movements and spatial positions. It can continuously record changes in brain activity in the natural behavioral flow, expanding the research scenarios from highly controlled laboratory environments to real-life situations, significantly enhancing the external validity and clinical translational value of the research results [8, 24]. fNIRS can stably capture the brain blood oxygen response characteristics in real scenarios such as natural social interactions, daily activity execution, classroom learning, and bedside assessment, providing reliable technical support for analyzing brain mechanisms of cognitive processing, social interaction, and emotion regulation in natural contexts. The rapid development of portable and wearable fNIRS systems has further promoted the application of this technology in real-world research, becoming an important technical link connecting basic experimental research and real-world applications [8]. At the same time, the non-invasive nature, radiation-free feature and low requirement for the subject's cooperation of fNIRS make it the preferred technique for brain function research in special populations such as infants and toddlers, the elderly, patients with consciousness disorders and those with implanted metal devices [25, 44]. In the field of pediatrics, fNIRS can conduct brain function tests for key developmental periods such as facial perception, emotional touch sensation, and language processing in infants in a state of wakefulness and naturalness, without the need for sedation or head fixation [32]. In elderly and patients with neurodegenerative diseases, fNIRS also demonstrates excellent tolerance and data acquisition success rate [45, 46]. Furthermore, the miniaturization and portability of fNIRS equipment enable its flexible deployment in bedside, rehabilitation center, and community settings, significantly enhancing the clinical accessibility of brain function assessment [24, 47]. fNIRS has the capability of sampling at a millisecond level (10–100 ms), and can capture the hemodynamic changes during task execution or intervention processes in real time, providing immediate feedback for clinical treatment [22]. This feature holds significant value in contexts such as neurorehabilitation, psychotherapy, and surgical monitoring. For instance, in stroke rehabilitation training, fNIRS enables real-time monitoring of motor cortex activation in patients, providing an objective basis for the dynamic adjustment of rehabilitation protocols [25]; During the perioperative period, fNIRS can continuously monitor cerebral oxygen saturation, helping anesthesiologists detect and intervene in cases of cerebral hypoxia in a timely manner, thereby reducing the risk of postoperative cognitive dysfunction [48, 49]. This real-time monitoring capability makes fNIRS not only a research tool, but also an auxiliary means for clinical decision-making. fNIRS has good complementarity with technologies such as EEG and fMRI. fNIRS can make up for the spatial positioning deficiency of EEG, while EEG can supplement the time resolution limitation of fNIRS in neural electrical activity. The integration of the two can simultaneously obtain neuroelectrophysiological and hemodynamic information, enhancing the comprehensive analytical ability for brain functional status [50].

**Fig. 3:**
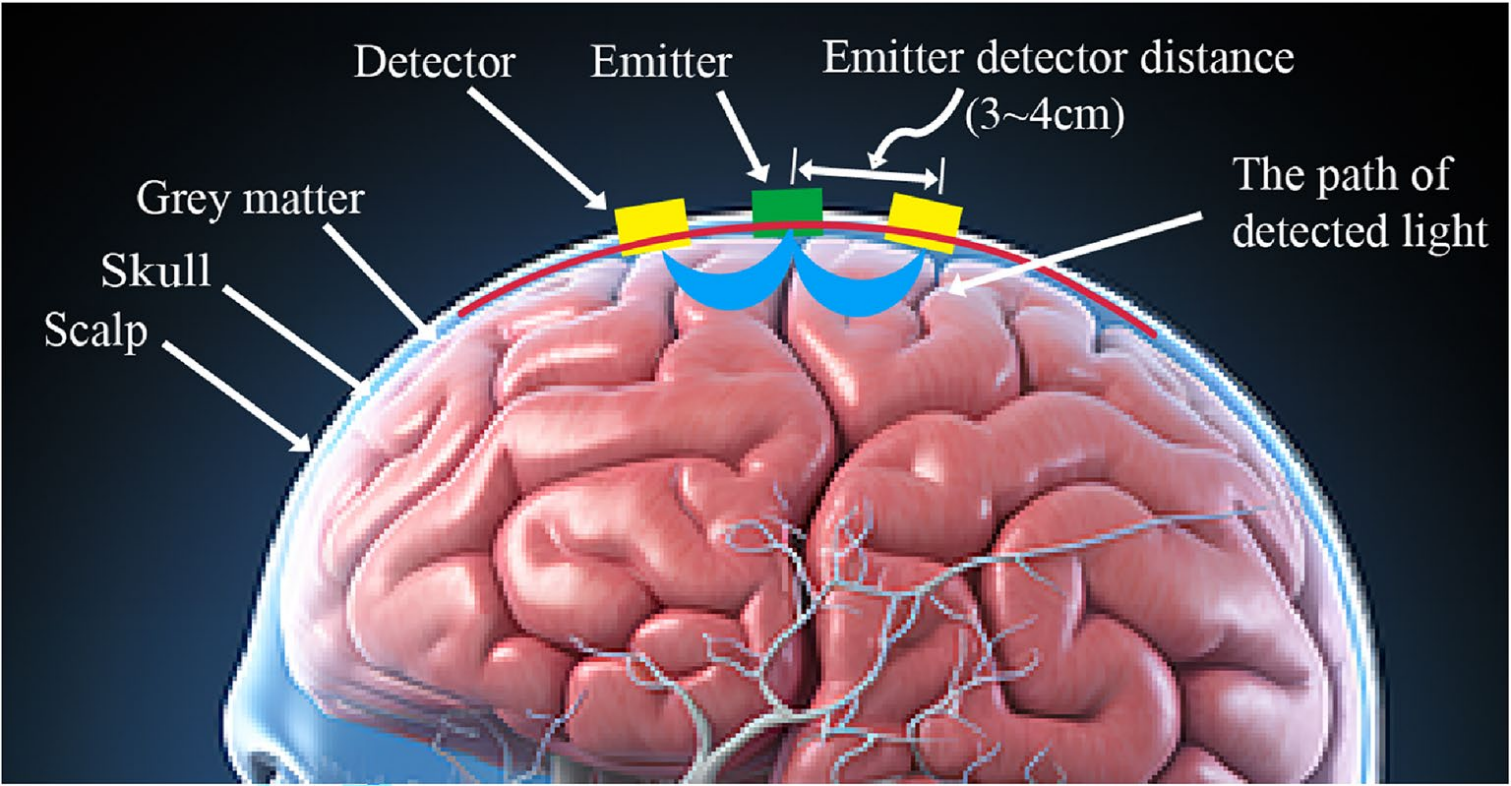
Functional near-infrared emitter-detector

The combined application of fNIRS and fMRI can establish a bridge between natural contextual tasks and high-precision structural imaging of the entire brain, enabling multi-scale observations ranging from macroscopic brain networks to local cortical activities [28]. Furthermore, the compatibility of fNIRS with metal implants enables its combined application with technologies such as transcranial magnetic stimulation (TMS), allowing for synchronized intervention and monitoring, and providing a powerful tool for the study of neural regulatory mechanisms [31, 50]. In summary, leveraging its unique advantages in ecological validity, applicability to special populations, real-time monitoring capability, and multimodal integration, fNIRS has gradually evolved from a basic research tool into an important technological platform for clinical assessment and intervention monitoring in the medical field. These advantages lay a solid foundation for its broad application in the diagnosis of neuropsychiatric disorders, evaluation of rehabilitation efficacy, investigation of mechanisms underlying TCM, and personalized medicine (Fig. 3).

### Applications of neuroscience and brain function research

Leveraging its non-invasive nature, portability, and high tolerance to motion artifacts, fNIRS has become an important tool for research on cognition, emotion, and the social brain. However, existing studies still face limitations such as high methodological heterogeneity, inconsistent task paradigms, small sample sizes, and insufficient reproducibility; consequently, some findings require further validation through large-sample, longitudinal, and cross-cohort studies. In this section, while summarizing key findings, we provide a critical review of research limitations, conflicting evidence, and methodological challenges, aiming to inform the development of standardized protocols in future research.

fNIRS has been widely applied in cognitive tasks involving working memory, inhibitory control, attention, and executive function. Classic N-back task studies have shown that as task load increases, oxyhemoglobin (Oxy-Hb) levels in the dorsolateral prefrontal cortex (DLPFC) of healthy individuals rise significantly, suggesting the involvement of the prefrontal cortex in cognitive resource allocation [38, 74]. However, the reproducibility of such findings varies considerably across different ages, genders, baseline abilities, and task parameters. Some studies have failed to replicate load‑dependent prefrontal activation, likely due to differences in channel placement, baseline correction methods, motion artifact removal strategies, and variations in participant head circumference and skull thickness. Moreover, most studies rely on group-level averaging, which limits generalizability to individual diagnosis; the insufficient signal-to-noise ratio at the single-subject level remains a critical bottleneck for clinical translation. In studies on mild cognitive impairment (MCI) and aging, fNIRS has revealed abnormally reduced prefrontal activation in patients during cognitive tasks, suggesting its potential as an early screening marker [51]. However, existing evidence remains inconsistent: some studies report compensatory increases, while others show decreased function. These discrepancies may arise from differences in task type, baseline cognitive level, data analysis strategies, and whether extracerebral blood flow interference has been removed. Therefore, although fNIRS holds potential as a screening tool, it cannot yet serve as a standalone diagnostic modality, and the establishment of unified paradigms and normative data is urgently needed.

In the field of social and developmental neuroscience, fNIRS has overcome the strict limitations imposed by traditional neuroimaging techniques such as fMRI and PET, which require subjects to be highly cooperative and maintain a fixed position. This is due to its unique advantages of being non-invasive, radiation-free, immune to movement interference, and capable of detecting in natural environments. Particularly suitable for special populations such as infants who are difficult to examine with conventional methods, fNIRS has become a core technique for exploring the neural mechanisms underlying early social cognition and affective processing. A series of fNIRS-based studies have systematically revealed the brain developmental trajectories of key social cognitive abilities, including infant face perception, affective touch processing, and social attachment. Regarding the development of face perception, studies have found that at 5–8 months of age, infants showed a specific hemodynamic response in the right temporal lobe when observing upright faces, whereas inverted faces do not evoke significant activation, suggesting right‑hemisphere dominance in early configural face processing. By 7–8 months of age, infants can recognize structured faces (e.g., Arcimboldo images) as faces, accompanied by left temporal lobe activation, marking a critical developmental period for holistic face processing at 7–8 months [52]. Further investigations into the maturation of face perception indicate that 5–8 months is a key phase for the development of view‑invariant face processing: 8-month-old infants show specific activation in the right temporal lobe in response to both frontal and profile views of faces, whereas 5-month-old respond only to frontal views. Meanwhile, by 7–8 months, infants exhibit bilateral temporal lobe neural adaptation effects for face identity invariance across non-rigid changes such as different expressions or mouth movements, whereas no such adaptation is observed at 5–6 months, delineating a critical developmental window for facial identity processing [53–55]. Cross-cultural studies further confirm the cross-cultural universality of face perception narrowing. Japanese infants can distinguish between adult and infant faces at 3 months of age, but by 9 months they show specific discrimination ability only for adult faces, with the right temporal lobe involved in configural processing of adult faces, reflecting the developmental principle of perceptual specialization driven by social experience [56]. At the level of affective processing and social interaction, studies have established that the cortical circuitry for affective touch undergoes critical development between 6 and 10 months of age. Specifically, 10-month-olds exhibit bilateral prefrontal cortex activation in response to gentle touch, whereas no such response is observed at 3–6 months [57]. In the context of mother-infant attachment, mothers viewing their own infant's smiling face show significant activation in the anterior orbitofrontal cortex bilaterally, and the activation intensity is positively correlated with positive emotion ratings. At 12 months of age, when observing their mothers' smiling faces, infants also showed specific responses in the medial prefrontal cortex and the orbitofrontal cortex. This suggests that the orbitofrontal cortex is a core brain region for encoding maternal-infant attachment and processing positive emotions, and this neural circuitry has already achieved mature functionality around the age of 1 year [58]. In the field of social affective neuroscience, fNIRS studies have demonstrated that gentle velvet stroking on the left palm significantly increases oxygenated hemoglobin levels in the frontopolar cortex (FPC) and orbitofrontal cortex (OFC), and that signal intensity is positively correlated with subjective pleasantness, thereby providing non‑invasive, highly ecologically valid objective evidence for the central mechanisms underlying socio‑affective tactile processing. Building on this, fNIRS has been further applied to research on grandparental attachment, revealing that when grandmothers watch videos of their grandchildren, significant activation is observed in the FPC, medial prefrontal cortex (mPFC), and DLPFC, with activation levels significantly correlated with subjective ratings of love. Notably, smiling expressions further enhance activation in the medial and superior prefrontal cortex, suggesting that the neural mechanisms of grandmotherly love involve the synergistic interplay of reward monitoring, theory of mind processing, and cognitive control [59, 60].

fNIRS can safely and stably capture cortical hemodynamic features of infant social cognition and emotional processing, providing objective biomarkers for elucidating how early social experiences, face perception, and affective touch shape brain development. This fully demonstrates its irreplaceable value in populations that have difficulty complying with traditional neuroimaging protocols, such as infants and the elderly. Furthermore, fNIRS reliably captures cortical hemodynamic responses to socio-affective and attachment behaviors under naturalistic conditions, offering an objective, quantifiable, non-invasive neuroimaging tool for investigating the central mechanisms underlying human emotional experience, tactile reward, and prosocial behavior. In doing so, it has significantly advanced the ecological and developmental directions of social and developmental neuroscience. However, it is noteworthy that fNIRS studies in this field generally suffer from small sample sizes and a lack of longitudinal follow-up. Moreover, substantial variability in stimulation materials, frequency, and baseline length across laboratories hampers direct comparability of results.

The core value of fNIRS in the field of emotion and social research lies in its ability to stably measure brain activity in natural interactions, observation of facial expressions, verbal communication, and real stress situations, which is something that traditional fMRI/EEG cannot achieve. Compared with EEG, fNIRS provides more precise cortical hemodynamic localization; compared with fMRI, fNIRS allows face-to-face interaction, free expression, natural speech, and body movement, thereby enabling the investigation of ecologically valid patterns of emotional response. For example, using fNIRS under naturalistic stress conditions (e.g., public speaking or examinations), studies have confirmed that prefrontal Oxy-Hb changes are quantitatively correlated with subjective anxiety and the use of emotion regulation strategies, offering an objective measure of regulatory effectiveness [48]. Other studies have shown that when participants view faces displaying different emotions, prefrontal cortex activity patterns exhibit significant differences in emotion recognition, particularly in response intensity when processing angry versus happy faces [61]. Furthermore, fNIRS has been employed to investigate the impact of self-disclosure on social interaction, revealing that sharing negative experiences significantly enhances emotional resonance and neural synchrony among participants, underscoring the important role of emotional expression in facilitating social behavior [62]. Collectively, these studies demonstrate that fNIRS not only elucidates the neural underpinnings of emotional processing but also offers new perspectives for understanding social behavior, thereby promoting mental health and social competence. Nevertheless, limitations in this field remain, including substantial variability in emotional stimulus paradigms, inconsistent baseline correction methods, susceptibility to motion artifacts from facial movements, a lack of longitudinal and interventional evidence, and an inability to probe subcortical emotion centers.

In studies of decision-making and cognitive load, fNIRS has provided neural evidence supporting classical theories such as cognitive resource theory, dual-process theory, and executive function models. According to cognitive load theory, high-load tasks consume limited working memory resources, leading to increased prefrontal cortex activation. fNIRS studies have shown that during decision-making under high cognitive load, activity in prefrontal and parietal networks is significantly elevated, reflecting the engagement of inhibitory control, conflict monitoring, and rational reasoning processes [63, 64]. However, existing studies still have notable limitations. For instance, most tasks are laboratory‑based simplified decisions that lack ecological validity; there is insufficient exploration of higher‑order processes such as risky decision‑making, intertemporal choice, and social decision‑making; and few studies have distinguished the neural contributions of cognitive load, emotional load, and motivational conflict. Furthermore, findings regarding the functional dissociation of prefrontal subregions remain inconsistent across studies, highlighting the need for finer anatomical localization and larger-sample validation.

fNIRS can be used to monitor brain functional plasticity changes before and after psychological interventions, with prefrontal hemodynamic alterations being significantly correlated with symptom improvement. By analyzing fNIRS data under different emotional states, researchers can identify brain activity patterns in regions associated with affective disorders such as anxiety and depression, thus informing mental health interventions [65]. Furthermore, fNIRS can be used to assess the efficacy of psychological treatments by evaluating their neural impact. Studies have demonstrated that after undergoing psychotherapy, patients show significant improvements in prefrontal Oxy-Hb levels during emotion regulation tasks, which correspond to reductions in their emotional symptoms [66]. However, existing intervention studies generally suffer from small sample sizes, lack of control groups, short follow-up periods, and an absence of established standardized predictors of treatment response.

In recent years, fNIRS has provided important objective evidence for the identification of neural biomarkers across a range of neuropsychiatric disorders, cognitive states, and physiological intervention settings. First, in terms of task-state monitoring of cortical functional specificity, fNIRS has been used in conditions such as developmental coordination disorder, major depressive disorder, and upper limb dystonia. Using standardized tasks including verbal fluency, finger tapping, and n-back, fNIRS reliably captures abnormal Oxy-Hb activation in key regions such as the prefrontal cortex, temporal lobes, and sensorimotor cortex in patient populations [67–69]. These task-driven activation deficits are not only correlated with the core cognitive-motor impairments of the respective disorders but also show graded changes with task complexity, providing direct evidence for the development of task-dependent biomarkers. Second, in the assessment of resting-state hemodynamic functional connectivity and network stability, studies on disorders of consciousness, stroke, and shift work fatigue have revealed significantly reduced functional connectivity in frontoparietal regions, motor-related cortices, and interhemispheric connections in patients or under special physiological states. Resting-state fNIRS has also validated the test–retest reliability of low-frequency hemodynamic measures in stroke patients. These findings indicate that resting-state functional connectivity can serve as a stable biomarker for evaluating brain network integrity, rehabilitation potential, and cognitive fatigue states [39, 41, 70]. Third, in terms of multimodal integration to enhance biomarker sensitivity and specificity, the combined use of fNIRS with EEG and fMRI has demonstrated complementary advantages in seizure detection and stroke rehabilitation assessment. The hemodynamic information provided by fNIRS compensates for the limitations of EEG in spatial localization and assessment of vascular reactivity, whereas the high temporal resolution of EEG complements fNIRS in terms of neural electrical source localization. Their integration has significantly improved diagnostic classification accuracy (e.g., 89.7% sensitivity and 95.5% specificity for seizure detection; 87.9% accuracy for differentiating minimally conscious state from unresponsive wakefulness syndrome) [37, 71]. Fourth, regarding dynamic monitoring under physiological and pharmacological intervention conditions, fNIRS can sensitively capture the acute modulatory effects of dietary bioactive components such as caffeine and resveratrol on prefrontal hemodynamics. It can also quantify in real time the dynamic hemodynamic changes during the maintenance and emergence phases of anesthesia as well as under working memory load. This ability to quantify the differences in states before and after intervention enables it to demonstrate unique potential for biomarker screening in the fields of anesthesia depth assessment, cognitive fatigue monitoring, and nutritional neuroscience [38, 72, 73].Further studies have shown that when infants engage in three-party joint attention interactions, fNIRS data exhibit specific activation in the left dorsolateral prefrontal cortex, and this effect is only significant under the condition of complete joint attention, without such an effect in the absence of a reference object or eye contact. This suggests that human infants already possess the neural basis for supporting joint attention in the early stage. The left dorsolateral prefrontal cortex is the core brain region for infants' social cognition and joint attention processing, providing objective evidence for the neural origin of human social and cultural learning [74, 75]. In summary, fNIRS provides a high-ecological-validity tool for neuroscientific research on cognition, emotion, and social and social brain processes. However, its findings are still influenced by methodological heterogeneity, localization accuracy, motion artifacts, and individual differences. Future research should establish unified paradigms, standardized preprocessing pipelines, large-sample validation, multi-center replication, and integrate multimodal data to enhance reliability. The true value of fNIRS lies not merely in "measuring activation" but in revealing, within naturalistic settings, patterns of real brain activity that other techniques cannot capture (Table 2).

**Table 2:** fNIRS in the field of neuroscience and brain function-summary of classic experimental paradigms

Paradigm category	Sample capacity	Experimental paradigm	Main discovery	References
Face processing and inversion effect	10 (adult)	Present upright/inverted faces, and record the changes in oxygenated hemoglobin in the frontal lobe and temporal lobe	The upright face evokes stronger bilateral temporal lobe activation, proving that face processing is specific	[53]
The development of infant face perception	12 (5–8 month old infants)	Face vs non-face visual stimuli: Comparison of brain region activation differences	Infants begin to exhibit specific temporal lobe activation for faces from the age of 5 months, reflecting perceptual narrowing	[55]
Emotional face processing	15 (adult)	Visual presentation of happy/angry/neutral facial expressions, measuring the orbitofrontal cortex and the prefrontal lobe	Emotional faces preferentially activate the orbitofrontal cortex, and negative faces elicit stronger responses	[58]
n-back	20 (adult)	0-back/1-back/2-back Task	The higher the task load is, the stronger the activation of the DLPFC will be	[38]
Face invariant across viewpoints	11 (7 month old infants)	Repeatedly presenting faces from different perspectives, using the neuroadaptive paradigm	Seven-month-old infants have developed invariant neural representations of faces across different viewpoints	[52]
Social interaction	13 (8–12 month old infants)	Interactive eye contact vs Non-eye contact	Joint attention significantly activates the superior temporal sulcus and the medial prefrontal cortex of infants	[74, 75]

### Clinical medical applications

Studies indicate that fNIRS can monitor changes in cerebral blood oxygen levels in real time, thereby evaluating the recovery of brain function in stroke patients. By observing oxygen saturation in specific brain regions, clinicians can more accurately assess patients' rehabilitation progress. For example, one study found a significant correlation between motor cortex activity monitored by fNIRS and rehabilitation outcomes in stroke patients, providing a basis for individualized rehabilitation programs [20]. Additionally, the non-invasive nature and portability of fNIRS make it easy to implement in clinical settings, offering patients a more comfortable monitoring experience. In the management of epilepsy, fNIRS technology also demonstrates unique advantages. By monitoring real-time changes in cerebral blood oxygenation during epileptic seizures, fNIRS can assist physicians in identifying seizure types and frequency, thereby enabling the development of more effective treatment plans. Studies have shown that fNIRS combined with machine learning algorithms can effectively distinguish epileptic seizures from other non-epileptic events, providing more accurate clinical diagnostic support [77, 78]. In the evaluation and monitoring of neurodevelopmental disorders, fNIRS technology offers new possibilities for early identification and intervention. Studies have shown that fNIRS can effectively monitor brain activity in children with autism spectrum disorder (ASD) during social interactions, revealing significant differences in prefrontal cortex activity compared to typically developing children when processing social scenarios [79]. According to fNIRS data, high-risk ASD individuals exhibit abnormal activation in brain regions related to social visual or auditory stimuli, such as certain temporal lobe areas (e.g., superior temporal sulcus and superior temporal gyrus). These regions appear to lack differentiated responses to social versus non-social stimuli, which may underlie one of the neural bases for their social cognitive deficits [80]. However, the results of such studies should be interpreted with caution, as the existing evidence has certain limitations. Most studies have small sample sizes, exhibit substantial variability in task paradigms and stimulus settings, and lack standardization in data preprocessing methods, leading to insufficient reproducibility and cross-center comparability of the findings. Furthermore, fNIRS can only detect hemodynamic changes in the superficial cerebral cortex and cannot capture ASD-related abnormalities in subcortical nuclei or brain network connectivity. Therefore, it is not yet able to fully elucidate the neuropathological mechanisms underlying ASD. fNIRS data have shown that individuals at high risk for autism exhibit abnormal activation in relevant brain regions when processing social visual or auditory stimuli. Specifically, temporal regions such as the superior temporal sulcus and superior temporal gyrus fail to show differential responses to social versus non-social stimuli, which may represent an important neural basis for their social cognitive deficits.

In recent years, fNIRS has also been gradually applied to research on attention-deficit/hyperactivity disorder (ADHD). For example, it has been used to compare brain activity between healthy individuals and ADHD patients for disease characterization [81], distinguish between different ADHD subtypes or presentations [82], and dissociate various forms of executive dysfunction [83].In a study employing simultaneous fNIRS-EEG fusion technology, fNIRS activation maps were used as spatial priors for EEG source localization to investigate dynamic changes in brain networks in patients with mild Alzheimer's disease (AD) during a digit span task. The results revealed significantly reduced connectivity in the orbitofrontal and parietal cortices in the high-alpha and beta bands in the mild AD group. Across all frequency bands, the AD group exhibited significantly decreased node degree and clustering coefficient in the frontal pole and medial orbitofrontal cortex, while these metrics were significantly increased in the superior temporal sulcus, indicating characteristic abnormalities in brain network topology in AD. This approach validates that fNIRS can effectively enhance the accuracy of EEG source localization, providing a feasible solution for studying the network mechanisms of AD and for portable neuroimaging assessment [84]. Additionally, fNIRS has been employed to assess brain function in patients with MCI. Research has found distinct cerebral blood flow responses in MCI patients during cognitive tasks compared to healthy controls, providing potential biomarkers for early MCI detection [51]. By monitoring brain activity in patients with various neurodevelopmental disorders, fNIRS not only helps identify potential neurodevelopmental issues but also supports the development of personalized intervention strategies. These findings underscore the importance of fNIRS in the field of neuroscience, particularly its potential applications in pediatric and adolescent populations, but the relevant conclusions are still in the exploratory stage and require further verification through large sample sizes, longitudinal tracking, and standardized paradigms.

Over the past few decades, fNIRS has gradually emerged as a potential alternative functional neuroimaging technique for the early identification and progression monitoring of neurodegenerative diseases such as Alzheimer's disease (AD) and Parkinson's disease (PD). By comparing fNIRS data between AD patients and healthy individuals, studies have revealed reduced activation in the dorsolateral prefrontal cortex during verbal fluency tasks, decreased region-specific activation [45], as well as hypoactivation in the frontoparietal regions and superior temporal gyrus [46]. During digit span tasks, AD patients showed lower activation in frontal areas (frontopolar and orbitofrontal regions) [45]. In finger-tapping span tasks, progression from mild to moderate or severe AD was associated with gradually diminished activation in the frontal and bilateral parietal cortices [85]. During visuospatial working memory tests, AD patients exhibited less pronounced bilateral prefrontal activation, with minimal increase in activation as task difficulty escalated [86]. Similarly, comparisons between PD patients and healthy controls using fNIRS data demonstrated higher levels of prefrontal cortex activation in PD patients [87]. However, existing findings exhibit marked heterogeneity. Some studies report reduced brain activation, whereas others observe compensatory increases, and the conclusions are not entirely consistent. This heterogeneity may arise from multiple methodological factors, including differences in task paradigms, varying levels of disease severity, differences in medication status, inconsistent data preprocessing pipelines, and whether extracerebral blood flow interference has been effectively removed. Furthermore, applying fNIRS to populations with neurodegenerative diseases faces a series of methodological challenges: elderly patients often present with increased head movements, large individual differences in scalp and skull thickness, interference from underlying diseases and medications, and reduced signal quality stability. In addition, the limited spatial resolution of fNIRS makes it difficult to capture functional abnormalities in subcortical structures such as the hippocampus and basal ganglia, which are key affected regions in AD and PD. Therefore, fNIRS is currently better suited as an auxiliary tool for investigating mechanisms and monitoring treatment efficacy in neurodegenerative diseases, and it cannot yet serve as a standalone imaging basis for early or differential diagnosis. Through standardized fNIRS data acquisition and analysis, it holds promise for providing objective references for early screening, disease course monitoring, and personalized treatment of neurodegenerative diseases.

The human brain is one of the organs with the highest oxygen utilization rate, making it extremely vulnerable to hypoxic conditions [88]. Currently, numerous cerebral oxygen monitoring devices are widely used in cardiovascular surgery, carotid endarterectomy, shoulder arthroscopy, and other procedures [89]. These devices significantly reduce the incidence of postoperative mental disorders and delirium and improve the prognosis of patients [18, 90]. fNIRS technology enables real-time monitoring of cerebral oxygen levels during surgery, allowing surgeons to promptly assess patients' neurological status and adjust surgical strategies to minimize complications. For instance, in cardiac and neurosurgical procedures, fNIRS is employed to evaluate cerebral perfusion in real time, ensuring adequate oxygen supply to brain tissues during operations [48]. As a technique for monitoring regional tissue oxygen saturation, fNIRS facilitates early detection of cerebral and related tissue hypoxia, reduces anesthesia-related complications, improves perioperative prognosis, and accelerates postoperative recovery [76]. It can also assess noxious stimuli in various clinical settings, such as pain, cold, cutting, or burning, and measure sustained pain stimuli under awake or anesthetized conditions. For example, the dynamic changes in the condition (such as chronic osteoarthritis, persistent pain, intermittent triggered pain and progressive changes in neuropathic pain); the daily variation of pain; pain resistance and reactivity; evaluation of response ability; evaluation of placebo and non-placebo effects, etc.—these are practical applications. During surgery, realtime fNIRS monitoring of procedures that induce pain or nociceptive inflammatory responses (e.g., cutting, cauterization, traction) may reduce the need for postoperative analgesics and mitigate the progression of chronic postoperative neuropathic pain. Currently, an estimated 15–40% of surgical patients experience significant chronic postoperative pain. Although intraoperative monitoring based on near-infrared spectroscopy (fNIRS) has been recognized as having potential value in predicting chronic postoperative pain and assessing the efficacy of analgesic interventions, the current supporting evidence remains limited and preliminary. Therefore, further studies with larger sample sizes and standardized protocols are necessary to validate its clinical utility. Interventions targeting perioperative cerebral desaturation events (CDEs) have been shown to reduce the incidence of postoperative cognitive decline [49, 91]. Therefore, the way to reverse CDEs during the perioperative period is critically important. Realtime fNIRS monitoring enables therapeutic strategies to counteract cerebral desaturation, such as increasing cerebral blood flow and arterial oxygen content, deepening anesthesia depth, reducing morbidity or mortality in major organs, and shortening intensive care unit and hospital stays after coronary artery bypass grafting [49, 91–93]. In summary, fNIRS-based realtime monitoring during surgery and the perioperative period not only enhances treatment precision and alleviates patient suffering but also supports personalized therapy, underscoring its significant clinical value in surgical practice.

With the accelerating pace of modern life, increasing levels of stress have contributed to a growing incidence of mental disorders, among which schizophrenia and depression account for a significant proportion. By employing a general linear model with fNIRS to extract β-values representing the activation level of 52 channels in the prefrontal cortex of both healthy individuals and patients with schizophrenia, channels showing significant differences were selected to construct feature vectors for classifier training and classification. The final classification accuracy reached 88.15%, enabling automated identification of patients with schizophrenia [94]. In patients with depression, during the execution of language fluency tasks, there are significant differences in brain activation and brain functional connections compared to healthy controls in the dorsolateral prefrontal cortex. This suggests that the dorsolateral prefrontal cortex may be a specific brain region for differentiating between depression and healthy individuals [95]. Using fNIRS, the study investigated the cortical activation characteristics induced by limb acupuncture in patients with post-stroke hemiplegia. The results showed that acupuncture induced specific activation in the bilateral prefrontal and motor cortices. In patients with mild motor impairment, increased activation was predominantly observed in the motor areas of the unaffected hemisphere. In contrast, patients with severe impairment exhibited extensive bilateral activation (particularly in the affected hemisphere), with significantly higher response amplitudes. Moreover, the acupuncture-induced changes in cortical activation were negatively correlated with motor function in patients with mild impairment [96].In patients with schizophrenia, resting-state studies have shown hyperactivation of the default mode network and significantly reduced functional connectivity in the prefrontal cortex. During task-state conditions, specific hypoactivation is observed in core brain regions including the bilateral prefrontal cortex, temporal lobes, and temporoparietal junction. The degree of this abnormal activation is closely correlated with the severity of negative symptoms and impairments in social cognition (e.g., emotion recognition, theory of mind, and cooperative behavior). Notably, hypoactivation in the right superior temporal gyrus may serve as an early predictive marker for individuals at clinical high risk for psychosis. Functional near-infrared spectroscopy (fNIRS) can objectively capture hemodynamic changes in the brain following interventions such as antipsychotic medications, neuromodulation (tDCS/TBS), and neurofeedback. Combined with machine learning, fNIRS effectively aids in the auxiliary diagnosis and subtype differentiation of schizophrenia. Furthermore, fNIRS-based hyperscanning has revealed reduced inter-brain synchrony in the right inferior frontal gyrus during interpersonal cooperation in patients, providing neural circuit evidence for social cognitive deficits. Overall, fNIRS stably captures the aberrant brain functional network characteristics of schizophrenia, offering reliable objective indicators for elucidating pathological mechanisms, identifying robust biomarkers, and monitoring personalized treatment responses [97, 98].Using an fNIRS imaging genetics paradigm, a verbal fluency task was administered to a cohort of schizophrenia patients and healthy controls. The results showed that the polymorphism (rs35201266) of the EGR3 gene significantly modulated the oxyhemoglobin response in the left dorsolateral prefrontal cortex (DLPFC). Specifically, individuals carrying the AA genotype exhibited a significantly smaller increase in task-state oxyhemoglobin compared to those with the GG/GA genotype, and this effect was independent of disease diagnosis. Overall, schizophrenia patients showed significantly reduced prefrontal activation. Notably, only patients with the GG genotype demonstrated a positive correlation between the degree of prefrontal activation and the severity of positive symptoms, suggesting a possible compensatory activation in this genotype. These findings provide in vivo human evidence that EGR3 gene variation influences prefrontal hemodynamic function through neurodevelopmental pathways, offering objective imaging evidence for the pathological link between genetic susceptibility to schizophrenia and prefrontal hypoactivation. Furthermore, this study validates the reliability and utility of fNIRS as an imaging genetic endophenotype indicator [99]. Although fNIRS, as an emerging neuroimaging technology, can reflect substantial objective clinical information through data monitoring and can be applied in the auxiliary diagnosis of mental disorders such as depression and schizophrenia, it still holds great potential for exploring the pathophysiological mechanisms of mental diseases and evaluating treatment efficacy. Based on existing research and clinical applications, fNIRS can currently mainly assist researchers and clinicians in distinguishing healthy controls from patients with mental disorders. In the future, fNIRS should integrate advanced analytical methods such as machine learning and deep learning. By combining the strengths of traditional statistical approaches with modern algorithms, it is expected to realize feature re-extraction of fNIRS signals, explore potential biomarkers for depression, and develop auxiliary diagnostic and therapeutic tools for depression. These advances will help researchers and clinicians improve the diagnosis and treatment of depressive disorders [100] (Table 3).

**Table 3:** fNIRS in the field of clinical medicine-summary of classic experimental paradigms

Disease/Application scenario	Experimental paradigm	Main discovery	References
Stroke rehabilitation	Exercise task/Exercise imagination	Patients with depression show significant abnormalities in the activation and functional connectivity of the dorsolateral prefrontal cortex	[20, 96]
Epilepsy monitoring	Epileptic seizure period/inter-seizure period monitoring	fNIRS can detect changes in cerebral blood oxygenation related to seizures; combined with machine learning, it can distinguish between epileptic and non-epileptic seizures	[78]
AD	Word fluency task, digit span task, visual-spatial working memory task	The activation of the posterior dorsolateral prefrontal cortex, the frontal-parietal network, and the superior temporal gyrus in AD patients was significantly reduced; as the disease progressed, the activation showed a decreasing trend	[45, 46, 81]
PD	Complex walking tasks, cognitive tasks	The activation degree of the prefrontal cortex in PD patients was significantly higher than that in healthy controls when they were engaged in walking and cognitive tasks	[84, 87]
ASD	Social visual/auditory stimulation task, face processing task	Children with ASD show a lack of differentiated responses in the prefrontal cortex, superior temporal sulcus, and superior temporal gyrus to social stimuli	[80]
ADHD	Go/No-go task, executive function task	The hemodynamic response of the right prefrontal lobe in children with ADHD is abnormal, which can be used for subtype and executive function difference analysis	[78, 79, 81, 83]
MCI	Cognitive tasks, working memory tasks	The brain blood flow response patterns of MCI patients are different from those of healthy controls, and this can serve as an early indicator for identifying potential issues	[51]
Depressive disorder	Language Fluency Task	Patients with depression show significant abnormalities in the activation and functional connectivity of the dorsolateral prefrontal cortex	[49, 94, 96]
Schizophrenia	Activation extraction of the prefrontal pathway	Based on the signals from the prefrontal lobe, automatic patient identification can be achieved, with an accuracy rate of 88.15%	[94, 99]
Perioperative cerebral oxygen monitoring	Intraoperative real-time cerebral blood oxygen monitoring	Can monitor cerebral oxygen desaturation events and reduce the risks of postoperative cognitive impairment and delirium	[89, 90]

### Applications in TCM research

TCM syndromes represent the overall functional responses of the human body under pathological conditions, and they occupy a central position within the TCM theoretical framework [101]. In recent years, fNIRS, as a non-invasive brain function imaging method, has provided a new approach for exploring the central nervous mechanism of TCM syndromes. Taking the common liver depression syndrome in emotional disorders (depression) as an example, TCM syndrome differentiation mainly relies on subjective evaluation of symptoms, and the evaluation of therapeutic efficacy is easily influenced by individual subjective factors [102, 103]. fNIRS data have shown that patients with major depressive disorder (MDD) presenting the liver qi stagnation pattern exhibit specific cerebral blood flow abnormalities in the left dorsolateral prefrontal cortex (lDLPFC) and mPFC. Following treatment with selective serotonin reuptake inhibitors (SSRIs), hemodynamic activation in the lDLPFC improves significantly and remains stable for up to three months. The fNIRS signal changes precede clinical remission of depressive symptoms. Moreover, the concentration of Oxy-Hb measured by fNIRS can quantify prefrontal cortical activation, and the CH13 channel may serve as a sensitive indicator for monitoring treatment efficacy in MDD with the liver qi stagnation pattern [27]. However, existing studies lack an integrated interpretation of the pathogenesis of liver qi stagnation and brain functional abnormalities within the framework of TCM. Comparative investigations of fNIRS features across different TCM patterns are absent, and the quantitative diagnostic criteria for liver qi stagnation as well as the corresponding thresholds for fNIRS indicators have not been clearly defined. In addition, methodological limitations persist, including generally small sample sizes, lack of blinding and placebo controls, and insufficient control for the effects of concomitant medications and underlying diseases. Consequently, objective diagnosis of the liver qi stagnation pattern cannot yet be achieved. In a study on primary biliary cholangitis with cognitive impairment, fNIRS results showed that patients with deficiency pattern constitution had lower brain activation levels than those with excess pattern constitution, which is consistent to some extent with the TCM theory of “liver-kidney deficiency and marrow-brain malnutrition” [95]. Nevertheless, the association between TCM constitution and brain function remains an exploratory finding. Standardized classification criteria for constitution types are lacking, and confounding factors are insufficiently controlled. The causal relationship and underlying neural mechanisms between constitution and brain function still require longitudinal validation. Overall, fNIRS can provide visualizable evidence for investigating the central mechanisms of TCM patterns and constitutions, but a quantitative system corresponding one-to-one with TCM pattern differentiation criteria has not yet been established. Its clinical translation still requires support from standardized and normalized research.

As a traditional Chinese therapy, acupuncture facilitates the treatment and recovery of many neurological disorders without pharmacological intervention. However, the primary mechanisms underlying its effects on various diseases remain unclear. In recent years, fNIRS has been progressively used to monitor the impact of acupuncture on brain neural activity, thereby helping to elucidate its therapeutic mechanisms. For example, fNIRS has been employed to investigate the effects of acupuncture on cortical activation in stroke patients with hemiplegia. Results indicate that acupuncture can induce increased cerebral blood oxygenation in the bilateral prefrontal cortex and motor-related cortical areas in these patients [99]. Additionally, fNIRS has been applied to study the recovery mechanisms in mild cognitive impairment, contributing to the treatment of AD [104, 105] . Meanwhile, fNIRS also has increasingly widespread and in-depth applications in monitoring the hemodynamic changes of diseases such as attention deficit/hyperactivity disorder, neurogenic tinnitus, hypertension, insomnia, and biliary colic in children treated with acupuncture therapy [106-111] These studies not only revealed the mechanism of acupuncture therapy on the central nervous system, but also provided an objective neuroimaging basis for the individualized treatment plans of acupuncture therapy. Furthermore, fNIRS is not only used for monitoring the therapeutic process of acupuncture in disease treatment but also integrates the traditional acupoint efficacy from TCM with modern brain functional imaging, thereby fostering a connection between TCM theory and neuroscience. In a study utilizing fNIRS to monitor changes in oxygenated hemoglobin concentration in the prefrontal cortex during magnetic stimulation of the Shenmen acupoint (HT7), it was found that by comparing cerebral blood oxygen signals between the resting state and the magnetic stimulation state, fNIRS provided objective neuroimaging evidence for the "sedative effect of the HT7". According to fNIRS data, stimulation of the HT7 can induce extensive activation in the prefrontal cortex accompanied by a decrease in blood oxygen levels. This reduction in blood oxygen may reflect weakened local neuronal activity or decreased metabolic demand, suggesting a reduction in cortical excitability. This provides direct physiological evidence for the hypothesis that "the sedative mechanism of the HT7 may be related to inhibiting prefrontal overactivity", while also supporting the theory that acupuncture stimulation at HT7 possesses calming and tranquilizing effects [112]. In a study investigating the effects of acupuncture at four acupoints-Baihui (GV20), Neiguan (PC6), Shenmen (HT7), and Taichong (LR3)-on cerebral cortical blood oxygen levels in insomnia model rats, fNIRS was utilized to monitor real-time changes in cortical blood oxygen concentration in the model group, sham acupuncture group, and acupuncture group under different light stimulation conditions. Based on the collected data, the prefrontal cortex and occipital lobe were identified as key abnormally activated brain regions in insomnia model rats. Furthermore, in this study, fNIRS data were also employed to construct cortical hemodynamic maps, which visually reflected the activation and inhibition states of brain activity in different experimental groups through color contrasts [113]. As a real-time monitoring tool for evaluating how acupuncture regulates brain function, fNIRS not only identifies key brain regions associated with insomnia but also assesses the therapeutic effects of acupuncture by visualizing corresponding cerebral hemodynamic responses. Moreover, it demonstrates how fNIRS can reveal the brain mechanisms by which acupuncture at traditional acupoints modulates insomnia from the perspective of neurovascular coupling theory, thereby advancing acupuncture research from the molecular level to the systemic brain functional level. In a study investigating acupuncture combined with upper limb rehabilitation robot-assisted training in stroke patients, fNIRS served as an assessment tool to evaluate the impact of Xingnao Kaiqiao acupuncture combined therapy on brain function. It was used to monitor real-time changes in blood oxygen concentration in the brain regions of stroke patients in both the experimental and control groups, with a particular focus on areas closely related to motor control, spatial cognition, and executive functions, such as the angular gyrus, prefrontal region, and dorsolateral prefrontal cortex. By integrating data analysis with functional improvement scores, it was found that acupuncture combined with upper limb rehabilitation robot training could enhance cerebral oxygenation and metabolism, promote neural plasticity, and subsequently improve motor and daily living functions. This provides physiological evidence that acupuncture can improve cerebral blood flow and facilitate neural repair. fNIRS offers unique advantages in studies integrating acupuncture and rehabilitation medicine, providing preliminary, visualizable, and exploratory hemodynamic evidence for the central effects of acupuncture. However, its reliability, specificity, and reproducibility still require methodological validation. Moreover, most acupuncture studies lack sham acupuncture controls and fail to standardize needling depth or manipulation techniques, making it difficult to rule out placebo effects. Additionally, the limited spatial resolution of fNIRS prevents the analysis of acupuncture effects on deep brain nuclei; thus, the neural mechanisms of acupuncture cannot yet be fully elucidated. The strength of fNIRS lies in its ability to perform real‑time monitoring of acupuncture in awake, naturalistic states, which more closely resembles clinical acupuncture scenarios compared to fMRI. Nevertheless, its use as an objective evaluation tool is still at a methodological development stage.

fNIRS also holds exploratory value in studies of herbal interventions. Following administration of Cangai volatile oil, an increase in HbO_2_ in the right frontal lobe was observed, suggesting that activation in this region is associated with emotion regulation and providing neuroimaging clues for its ameliorative effect on depressive symptoms [114]. However, it is noteworthy that such results merely reflect the effect of the substance on cortical blood flow and cannot be directly equated with pharmacological targets. Moreover, confounding factors such as olfactory stimulation and emotional arousal have not been excluded, so causal inferences should be made with caution. In studies on the perception of bitter substances in TCM, bitterness is an important component of the “four natures and five flavors” theory, and bitter constituents serve both as taste substances and active pharmaceutical ingredients [111]. Following a “spectrum-taste association” approach that combines chemical fingerprinting with sensory evaluation, one study used fNIRS to assist in screening the bitter components of Panax notoginseng (ginsenosides Rg_1_, Rb_1_, and Rd) [115, 116]. However, it must be clarified that fNIRS, as a cerebral functional imaging technique, does not directly detect chemical taste signals but rather measures hemodynamic responses in the brain’s taste and reward networks. Therefore, fNIRS is not used for structural identification of compounds but for validating the central taste perception responses elicited by bitter components. Although preliminary associations between component intensity and brain response have been established, existing studies have notable methodological flaws: they fail to control for confounding factors such as taste adaptation, oral sensation, emotion, and attention; they have not established a quantitative model of brain activation for bitter perception; and fNIRS cannot distinguish central representations of different taste qualities such as bitterness, astringency, or pungency. Hence, its use for screening bitter components remains exploratory and cannot replace physicochemical detection or sensory evaluation.

**Fig. 4:**
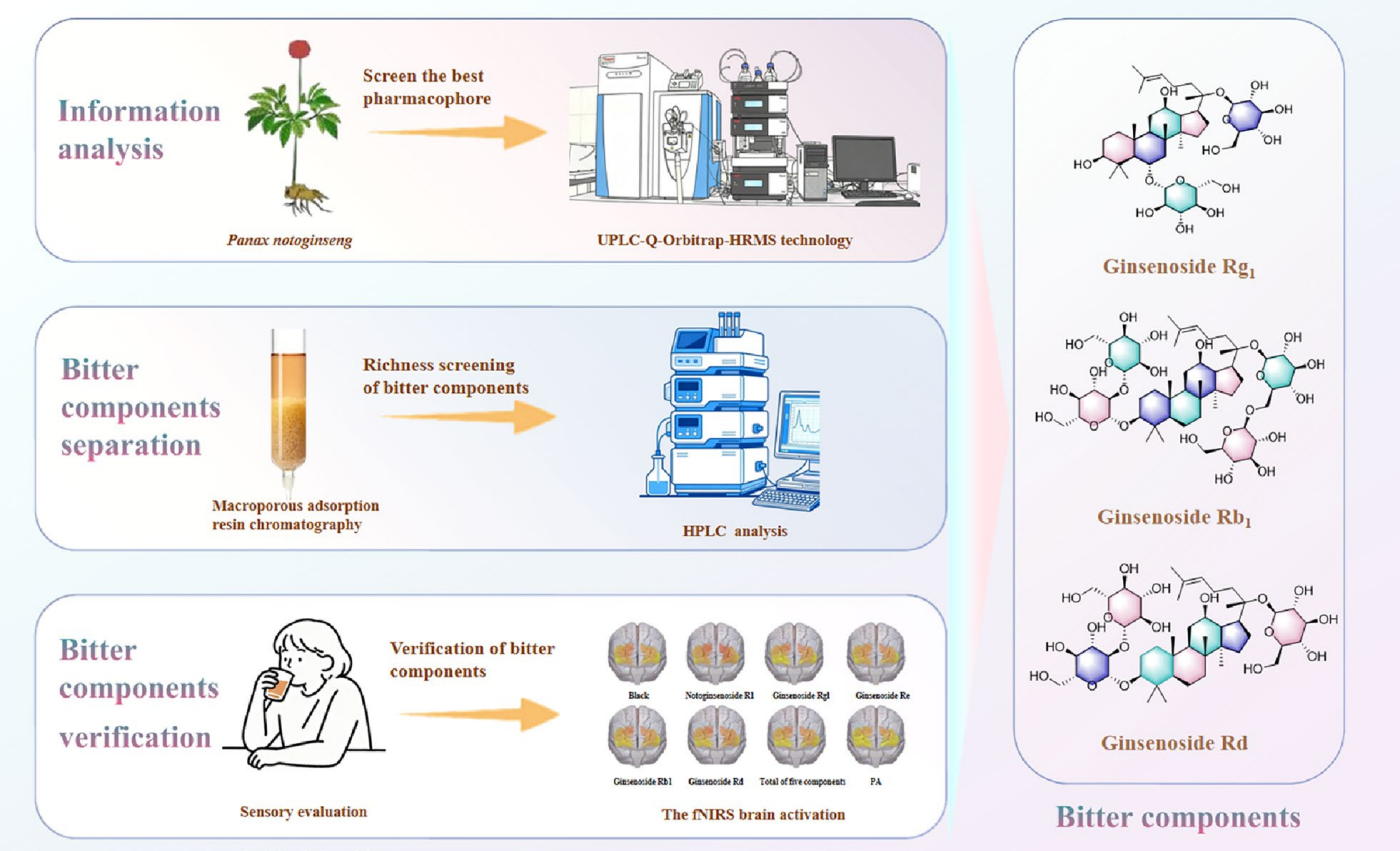
Discovery and verification of bitter components in Panax notoginseng based on the integrated strategy of pharmacophore model, system separation and bitter tracing technology

**Fig. 5:**
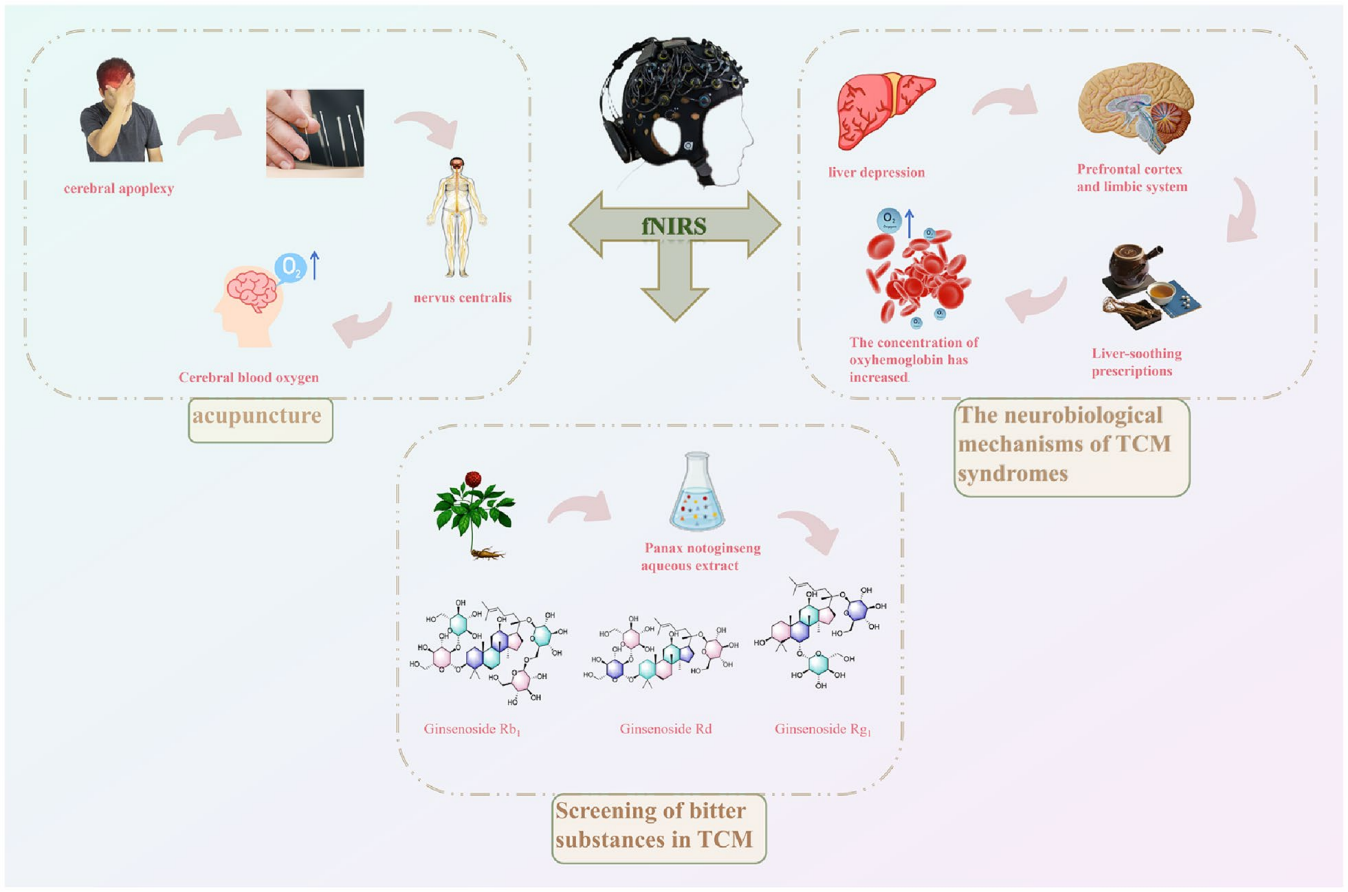
Applications in TCM research

In summary, fNIRS provides a non-invasive, ecologically valid visualization tool for investigating the central mechanisms underlying TCM patterns, acupuncture effects, and herbal interventions, thereby extending the objective study of TCM from the symptom level to the cerebral functional level. Nevertheless, its limitations must be objectively recognized. Current studies generally suffer from small sample sizes, lack of control groups, heterogeneous paradigms, insufficient reproducibility, and a lack of reliability data. fNIRS only reflects cortical hemodynamic changes and cannot be directly equated with the full biological basis of TCM pathogenesis. The associations between TCM theories and fNIRS indicators remain correlational findings and have not yet formed a generalizable, verifiable, and reproducible objective framework. Therefore, fNIRS should be regarded as an important exploratory tool for investigating TCM mechanisms, not as an independent diagnostic tool for clinical pattern differentiation or efficacy evaluation. Future research should establish standardized paradigms, conduct large-sample validation, incorporate multimodal integration, and rigorously control bias to enhance scientific rigor and translational potential (Figs. 4, 5) (Table 4).

**Table 4:** fNIRS in the field of TCM-Summary of classic experimental paradigms

Disease/Application scenario	Experimental paradigm	Main discovery	References
TCM syndrome (Liver depression syndrome—depression)	Emotional stimulation task, language fluency task, fNIRS comparison before and after treatment	Patients with liver depression syndrome showed significant reduction in HbO_2_ in the prefrontal cortex and limbic system; after the intervention of regulating liver and regulating qi, brain activation approached that of healthy individuals	[27]
Primary biliary cholangitis complicated with cognitive impairment	TCM constitution classification + Cognitive tasks	The brain activation level of patients with a deficiency constitution is significantly lower than that of patients with a solid constitution, which is consistent with the theory of "deficiency of liver and kidney and malnutrition of brain marrow"	[95]
Acupuncture intervention (for hemiplegia after stroke)	Acupuncture intervention + fNIRS monitoring of the motor-related cortex	Acupuncture can significantly increase the cerebral blood oxygen levels in both the bilateral frontal lobes and the motor cortex	[99]
Acupuncture intervention (for mild cognitive impairment)	Task-state fNIRS before and after acupuncture treatment	Acupuncture can improve cerebral blood flow dynamics and enhance the accuracy of classification recognition	[104, 105]
Acupuncture intervention (calming at the HT7)	Magnetic stimulation of the HT7, comparison between resting state and stimulated state	Stimulation of the HT7 reduces HbO_2_ in the prefrontal lobe, suggesting a decrease in cortical excitability, providing objective evidence for sedation and calming the mind	[112]
Acupuncture intervention (for insomnia)	Acupuncture at GV20, PC6, HT7 and LR3, along with cerebral cortex blood oxygen monitoring	The hemodynamic response of the right prefrontal lobe in children with ADHD is abnormal, which can be used for subtype and executive function difference analysis	[113]
Acupuncture combined with rehabilitation (for stroke)	Awakening and stimulating acupuncture method + Rehabilitation robot, fNIRS real-time monitoring	Joint intervention promotes regional oxygenation metabolism and neural plasticity in the brain	[116]
Depressive disorder	Language fluency task	Patients with depression show significant abnormalities in the activation and functional connectivity of the dorsolateral prefrontal cortex	[49, 94, 96]
TCM intervention (Cangai volatile oil)	Inhaling the volatile oil of traditional Chinese medicine, character/semantic fluency task	The HbO_2_ level in the right frontal lobe significantly increased, providing neuroimaging evidence for the alleviation of depression	[114]
Perception of the bitter taste in TCM (Panax notoginseng)	Taste stimulation of Panax notoginseng extract and fNIRS monitoring of brain perceptual response	Validation of ginsenosides Rg_1_, Rb_1_, and Rd as the major bitter-tasting constituents of Panax notoginseng	[115]

### Applications in rehabilitation medicine

The advantage of fNIRS in the field of rehabilitation medicine lies in its low requirement for the examination environment. It can be performed in various natural settings such as schools or hospitals, and even allows infants to be examined while held by parents. This makes it suitable not only for adults but also for infants and children with developmental disorders in brain function research [117]. In recent years, fNIRS has been progressively applied to the study of common pediatric rehabilitation conditions such as cerebral palsy, ASD, ADHD, Down’s syndrome, neonatal hypoxic-ischemic encephalopathy, specific language impairment, and epilepsy [118]. In adults, its applications focus more on rehabilitation training for sequelae including post-stroke aphasia, post-stroke depression, cognitive impairment, and motor dysfunction [117–119]. Among rehabilitation techniques, fNIRS is most frequently used in motor imagery therapy and repetitive transcranial magnetic stimulation (rTMS). Motor imagery therapy enhances motor function by repeatedly simulating and rehearsing movements in the brain without significant physical activity, utilizing motor memory to activate specific brain regions and repeat transcranial magnetic stimulation [120]. fNIRS data revealed that when patients imagined skilled upper limb activities, brain signal activation was more pronounced [121]. Studies on rTMS using fNIRS suggested that its analgesic mechanism in rehabilitation may be related to the inhibition of activation in the primary motor cortex and premotor cortex [122]. These findings indicate that fNIRS can serve as an important tool for evaluating the efficacy of rehabilitation technologies.

### Shortcomings and future directions of fNIRS technology

As a non-invasive optical imaging technique for brain function, fNIRS has been widely applied across various research fields owing to its advantages of portability, motion tolerance, and high ecological validity. However, it also has several drawbacks and limitations. The physical constraints and application shortcomings of fNIRS are mainly reflected in the following aspects. In terms of imaging principles, fNIRS only detects the superficial cortical gray matter with a spatial resolution of 1–3 cm. It cannot cover deep nuclei such as the basal ganglia or hippocampus, nor can it distinguish fine functional subdivisions of sulci and gyri [15, 123]. This physical limitation cannot be overcome by algorithmic compensation, which restricts its validity in studies of cognitive control, memory, and emotion that are dominated by deep brain structures; essentially, fNIRS remains a surface-cortical tool. Regarding signal quality, fNIRS is susceptible to extracerebral physiological noise. Although existing methods such as filtering, short-distance regression, independent component analysis, and deep learning can partially suppress noise, they rely on assumptions, involve complex parameters, and yield unstable results, making pure extraction of brain signals difficult [124]. Moreover, motion artifacts are difficult to eliminate; slight head movements, facial expressions, and limb movements during testing can cause optode displacement, leading to baseline drift and spike noise. This is particularly prominent in infant, clinical, and naturalistic settings and is the primary cause of data inaccuracy [125]. In quantitative capability, mainstream continuous‑wave devices only provide semi-quantitative measurements of relative concentrations. Individual differences in head shape, scalp thickness, skin tone, and hair characteristics significantly amplify measurement errors, making it difficult to use fNIRS as a quantitative diagnostic tool [126]. For spatial localization, fNIRS lacks structural imaging capability and must rely on MRI or MNI templates for registration, a process that is cumbersome and prone to error. Localization accuracy is further reduced in infant and pathological populations, resulting in insufficient reproducibility and interpretability [15]. In addition, the low signal-to-noise ratio at the single-subject level-characterized by weak and fluctuating signals-necessitates large sample sizes and group averaging to obtain stable results [127]. This severely limits applications such as individualized diagnosis, real-time brain-computer interfaces, and bedside rapid assessment. Inconsistent light transmission efficiency due to individual differences in hair, skin tone, and head circumference further compromises signal quality, making fNIRS findings poorly comparable across populations and laboratories [128]. Based on the above analysis, fNIRS has limitations in penetration depth, systemic physiological noise, and motion artifacts. These shortcomings not only constrain its validity in deep-brain functional research and clinical translation but also pose serious challenges in key scenarios such as single-subject analysis and cross-population comparability. Consequently, how to overcome these bottlenecks through hardware design, algorithmic frameworks, and multimodal integration has become a central issue for the future development of fNIRS. Given current technological trends, its possible future directions are discussed from several aspects.

The application of fNIRS in the medical field continues to expand. Its future development needs to be grounded in existing technological bottlenecks and published empirical research, advancing steadily in hardware optimization, algorithmic innovation, clinical translation, and multimodal integration. The core limitations of current fNIRS are its limited spatial resolution, susceptibility of signal‑to‑noise ratio to interference from the scalp and skull, insufficient signal specificity, and inability to probe deep brain structures. Therefore, technical improvements should focus on addressing these practical issues. Hardware and device development-Improving the spatial resolution and signal-to-noise ratio of fNIRS is an important direction. Studies have confirmed that using multi‑wavelength laser sources, high-density probe arrays, and short‑separation detector designs can effectively suppress extracerebral tissue interference and improve signal specificity [129]. However, such improvements are still at the laboratory stage and have not yet been translated into clinical general-purpose devices. Moreover, constrained by the physical penetration limits of near-infrared light, detection depth cannot exceed the superficial cortex; this physical limitation cannot be fully overcome by upgrades to light sources and detectors alone. Portable, wireless wearable fNIRS devices have demonstrated feasibility for naturalistic monitoring and can support out-of-hospital rehabilitation and long-term dynamic brain function assessment. Nevertheless, existing devices still face challenges in battery life, anti-interference capability, and data synchronization; their stability and accuracy require validation through large-sample real-world studies [130].

Traditional fNIRS analysis methods are susceptible to individual differences, motion artifacts, and heterogeneity in experimental paradigms, leading to insufficient reproducibility of results. In recent years, machine learning and deep learning methods have been progressively applied to fNIRS signal denoising, feature extraction, and classification of neuropsychiatric disorders. Empirical studies have shown that convolutional neural networks (CNNs) can automatically extract spatiotemporal features from fNIRS data and achieve better accuracy than traditional handcrafted feature methods in identifying depression, schizophrenia, and other disorders. Machine learning models such as support vector machines (SVM) and linear discriminant analysis (LDA) can also effectively extract task-related features for cognitive function assessment and auxiliary discrimination of depressive states [131]. Although AI methods offer advantages in feature learning and classification performance, the generally small sample sizes in fNIRS research pose a common problem. Deep learning models require large amounts of data, and direct application can lead to overfitting and poor cross-center generalization [132]. To address the small-sample challenge, existing studies have explored strategies such as transfer learning, data augmentation, and few-shot learning to optimize the deep learning training pipeline for fNIRS, improving model stability and generalizability with limited data [133]. In the long term, establishing multi-center, large-sample fNIRS datasets with standardized acquisition protocols and preprocessing pipelines remains a core prerequisite for ensuring reliable deployment of AI algorithms. In addition, fusing fNIRS with modalities such as EEG and fMRI can complement spatiotemporal resolution limitations and improve the comprehensiveness of neural activity interpretation. Multimodal fusion models have demonstrated significantly better performance than single modalities in epilepsy and stroke rehabilitation assessment. However, current challenges remain in cross‑modal data registration, temporal synchronization, and fusion algorithms, which require further refinement.

In the personalized medicine applications, leveraging its real‑time, non‑invasive, and repeatable monitoring characteristics, fNIRS holds potential for dynamic assessment and efficacy prediction. In neurorehabilitation, fNIRS can observe changes in brain functional activation during motor imagery and task training. Preliminary studies suggest that brain activation patterns may be used to predict rehabilitation outcomes and assist in adjusting training protocols [129], but unified and validated clinical decision‑making indicators have not yet been established. In mental health, fNIRS can monitor prefrontal hemodynamic changes during emotional tasks, providing objective process measures for psychotherapy [50]; however, longitudinal intervention studies are still lacking to confirm that it can improve treatment outcomes. In drug development and evaluation, based on the principle of neurovascular coupling, fNIRS can monitor cerebral hemodynamic changes following pharmacological interventions. Studies have used it to compare the effects of the Mongolian medicine Sugmule‑4 and estazolam on brain function in patients with primary insomnia [130], providing objective imaging markers for evaluating central drug efficacy. Nonetheless, there is currently insufficient evidence to support the direct use of fNIRS for individualizing drug dose optimization. fNIRS can serve as a non‑invasive tool for monitoring central drug effects and help reflect the relationship between pharmacological action and brain function regulation, but extensive pharmacokinetic‑neuroimaging joint studies are needed before it can guide precise dose adjustment. In terms of the integrated application of AI, Machine learning and deep learning can enhance the efficacy of fNIRS in disease classification and early warning. Studies have applied these methods to the auxiliary identification of Alzheimer’s disease, Parkinson’s disease, epilepsy, and schizophrenia, capturing subtle brain functional changes that are difficult to detect with traditional statistical methods [134]. However, problems such as poor model interpretability, low cross‑device/cross‑laboratory generalizability, and insufficient sample size still constrain clinical translation. Future integration of AI and fNIRS should focus on algorithmic approaches that better suit the characteristics of fNIRS data, such as few-shot learning, transfer learning, and explainable models, rather than relying solely on generic deep learning architectures. At the same time, AI-assisted fNIRS analysis should be regarded as a decision-support tool and cannot replace clinical diagnosis and professional evaluation.

In summary, the future development of fNIRS should address existing technical limitations by following a path of “hardware precision, algorithm standardization, multimodal integration, and clinical empirical validation.” It holds broad prospects in rehabilitation monitoring, drug evaluation, and auxiliary disease diagnosis. However, all technological outlooks must be built upon verifiable, reproducible, and quantifiable research evidence to truly advance the translation of fNIRS from a research tool to a precision medicine platform.

## Conclusion

As a non-invasive, portable, and ecologically valid neuroimaging technique, fNIRS has demonstrated irreplaceable value in the assessment of neuropsychiatric disorders, monitoring of cognitive function, evaluation of rehabilitation outcomes, and investigation of mechanisms in TCM. Compared with conventional techniques such as fMRI and EEG, fNIRS can stably acquire cerebral hemodynamic signals in naturalistic settings, during dynamic tasks, and in special populations, thereby providing an important objective basis for clinical assessment and the elucidation of central mechanisms.

This systematic review confirms that fNIRS has yielded key research evidence in three major areas. In the field of neuroscience and cognitive science, fNIRS reliably reflects activation patterns in the prefrontal cortex, temporal lobes, and motor cortex during cognitive, emotional, decision-making, and social interaction processes. In clinical medicine, fNIRS can be used for stroke rehabilitation, seizure monitoring, screening of neurodevelopmental and neurodegenerative disorders, and perioperative cerebral oxygenation and pain monitoring, with potential value for early warning and efficacy assessment. In the field of TCM, fNIRS provides visualizable and quantifiable neural evidence for the brain mechanisms underlying TCM patterns, acupuncture effects, and herbal interventions, advancing TCM diagnosis and treatment from symptom description toward objective brain-function characterization. At the same time, this review identifies the core methodological challenges that constrain the clinical translation of fNIRS. These include: non‑standardized optode placement and channel configuration, significant individual differences in head anatomy and scalp-skull thickness, signal contamination from extracerebral blood flow and motion artifacts, heterogeneity in experimental paradigms and baseline correction procedures, predominance of small‑sample studies leading to insufficient reproducibility, and lack of cross‑center unified data standards. These issues directly lead to discrepancies in the interpretation of cortical activation and hinder cross‑study comparability, representing the most prominent bottlenecks in the field.

Therefore, future research urgently needs to establish implementable and concrete standardized protocols, including: unified standards for optode placement and anatomical registration templates, standardized procedures for short-channel noise reduction and extracerebral blood flow correction, standardized task paradigms and data preprocessing pipelines, quality control metrics for both resting‑state and task-state data, and the construction of multi-center, large-sample shared databases. On this basis, advancing multimodal integration such as fNIRS-EEG can compensate for limitations in spatiotemporal resolution. Combined with wearable devices and AI algorithms, this will enable robust feature extraction and individualized prediction, ultimately enhancing the reliability and clinical utility of fNIRS as a biomarker platform. In summary, by virtue of its unique ecological and clinical advantages, fNIRS has become an important bridge linking basic neuroscience, clinical medicine, and the modernization of TCM. After addressing key issues such as methodological standardization, signal quality control, and multi-center validation, fNIRS is expected to become a generalizable and translatable core technology platform for precision medicine, rehabilitation assessment, and objective TCM research.

## Data Availability

No datasets were generated or analysed during the current study.

## Abbreviations

fNIRS: Functional Near-Infrared Spectroscopy
TCM: Traditional Chinese Medicine
HbO_2_: Oxygenated Hemoglobin
HbR: Deoxygenated Hemoglobin
fMRI: Functional Magnetic Resonance Imaging
PET: Positron Emission Tomography
EEG: Electroencephalogram
rCBF: Regional Cerebral Blood Flow
CBV: Cerebral Blood Volume
NIR: Near Infrared
tHb: Total Hemoglobin
BOLD: Blood Oxygen Level-Dependent
ICC: Intraclass Correlation Coefficient
TMS: Transcranial Magnetic Stimulation
Oxy-Hb: Oxyhemoglobin
DLPFC: Dorsolateral Prefrontal Cortex
MCI: Mild Cognitive Impairment
FPC: Frontopolar Cortex
OFC: Orbitofrontal Cortex
mPFC: Medial Prefrontal Cortex
ASD: Autism Spectrum Disorder
ADHD: Attention-Deficit/Hyperactivity Disorder
AD: Alzheimer's Disease
PD: Parkinson's Disease
CDEs: Cerebral Desaturation Events
lDLPFC: Left Dorsolateral Prefrontal Cortex
SSRIs: Selective Serotonin Reuptake Inhibitors
MDD: Major Depressive Disorder
rTMS: Repetitive Transcranial Magnetic Stimulation
MNI: Montreal Neurological Institute
CNNs: Convolutional Neural Networks
SVM: Support Vector Machines
LDA: Linear Discriminant Analysis
